# Bridging the Gap Between Accuracy and Efficiency in AI-Based Breast Cancer Diagnosis from Histopathological Data

**DOI:** 10.3390/cancers17132159

**Published:** 2025-06-26

**Authors:** Kuldashbay Avazov, Sabina Umirzakova, Akmalbek Abdusalomov, Zavqiddin Temirov, Rashid Nasimov, Abror Buriboev, Lola Safarova Ulmasovna, Cheolwon Lee, Heung Seok Jeon

**Affiliations:** 1 Department of Computer Engineering, Gachon University, Sujeong-Gu, Seongnam-si 13120, Republic of Korea; kuldoshbay@gachon.ac.kr (K.A.); sabinatuit@gachon.ac.kr (S.U.); akmaljon@gachon.ac.kr (A.A.); 2 Department of Computer Systems, Tashkent University of Information Technologies Named After Muhammad Al-Khwarizmi, Tashkent 100200, Uzbekistan; 3 Department of Digital Technologies, Alfraganus University, Yukori Karakamish Street 2a, Tashkent 100190, Uzbekistan; z.temirov@afu.uz; 4 Department of Artificial intelligence, Tashkent State University of Economics, Tashkent 100066, Uzbekistan; rashid.nasimov@tsue.uz; 5 Department of AI-Software, Gachon University, Sujeong-Gu, Seongnam-si 13120, Republic of Korea; abror1989@gachon.ac.kr; 6 Department of Information Technologies, Samarkand State University of Veterinary Medicine, Samarkand 140103, Uzbekistan; lola.safarova.81@inbox.ru; 7 Department of Computer Engineering, Konkuk University, Chungju 27478, Republic of Korea; hsjeon@ku.ac.kr

**Keywords:** breast cancer classification, multi-scale feature extraction, histopathological image analysis, stain normalization, digital pathology

## Abstract

Background/Objectives: Breast cancer diagnosis using histopathological images remains a critical yet challenging task in computational pathology due to overlapping visual features between benign and malignant tissues, inconsistencies in staining, and variations in magnification. The objective of this study was to design a lightweight yet high-performing deep learning model that bridges the gap between diagnostic accuracy and computational efficiency. Methods: We propose CellSage, a novel convolutional neural network (CNN) architecture enhanced with attention mechanisms. It integrates three core modules: a multi-scale feature extraction unit designed to capture both global and local tissue patterns; a depthwise separable convolution block for reducing computational load; and a Convolutional Block Attention Module (CBAM) to dynamically focus on diagnostically relevant regions. The model was trained and evaluated on the BreakHis dataset, using stain normalization (via contrastive augmentation modeling, CAM) and extensive data augmentation techniques. A patient-wise cross-validation strategy was employed to ensure robust generalization. Results: CellSage achieved 94.8% accuracy, a 0.93 F1 score, and a 0.96 AUC, while remaining compact at only 3.8 million parameters. It outperformed deeper and larger models such as ResNet-50, DenseNet-121, and Vision Transformers in terms of both predictive performance and computational efficiency. Ablation studies confirmed that multi-scale feature extraction and attention refinement were critical components. Conclusions: CellSage is an interpretable, reliable, and computationally lightweight system for breast cancer diagnosis using histopathological data. Its efficiency and low computational footprint render it an ideal candidate for real-time deployment on digital pathology platforms, particularly in environments with limited computational infrastructure.

## Simple Summary

Breast cancer is one of the most common and lethal cancers among women worldwide. Accurate diagnosis is essential; however, analyzing tissue samples under a microscope can be challenging, time-consuming, and susceptible to human error. To assist clinicians, we developed a novel computer model named CellSage, which employs artificial intelligence to determine whether breast tissue is healthy or cancerous automatically. A distinguishing feature of CellSage is its integration of multiple techniques to analyze tissue images from various perspectives, thereby emulating the approach of a pathologist who examines both macroscopic structures and fine cellular details. The model is lightweight, efficient, and accurate, making it suitable for real-time clinical use, including deployment on devices with limited computational capacity. The evaluation demonstrated that CellSage outperforms several existing models while utilizing fewer computational resources. These findings suggest that CellSage could enhance the speed and accuracy of breast cancer diagnoses, thereby offering potential benefits for both patients and healthcare providers.

## 1. Introduction

Breast cancer continues to be a leading cause of cancer death in women globally [1], with timely and correct diagnosis being instrumental to improving the clinical outcomes [2]. Breast cancer diagnosis still hinges on the histopathological inspection of tissue biopsies, as it provides critical morphological insights needed to distinguish between benign and malignant lesions [3]. However, this method is not without limitations. The manual interpretation of histological slides is time-consuming, prone to inter-observer variability, and often subject to subjective judgment, especially in borderline or morphologically ambiguous cases [4]. Additionally, the increasing diagnostic workload in pathology laboratories can lead to reader fatigue, further increasing the risk of oversight. These challenges underscore the need for automated, reliable, and assistive tools that can enhance diagnostic precision and support clinical decision-making. Nevertheless, manual interpretation of histopathological slides is subjectively biased [5], time-consuming [6], and fraught with inter-observer variability [7] in borderline or morphologically ambiguous cases [8]. These facts reveal the need for comprehensive automated tools that can aid in diagnostic precision and act as reliability enhancers for pathologists dealing with automated analysis systems. While traditional machine learning approaches relied on manually designed feature extractors, CNN-based models perform automatic feature extraction through stacked convolutional layers. In this study, we use the term “feature extraction unit” to refer to the learned representation layers within the CNN architecture, particularly the multi-scale convolutional module that captures tissue patterns at both macro and micro levels. Although manual feature engineering is no longer required, feature extraction remains a fundamental step in any deep learning pipeline.

The use of deep learning, especially convolutional neural networks (CNNs), has been extraordinarily effective in the completion of medical imaging processes such as detecting, segmenting, and classifying cancers [9]. CNN-based models have greatly surpassed traditional feature-extraction-based methods in computation, accuracy, and generalization by learning image features hierarchically, transforming the graphics data into formal and surpassing traditional methods [10]. However, two critical obstacles remain in applying deep learning models to clinical pathology. First, most high-performing CNN models contain a high degree of parallelism, posing a challenge to real-time execution in situations with limited computational resources [11]. Second, the inherent diversity of histopathological images concerning stain intensity scattered image parts, tissue structure, and magnification level not only requires accuracy but also model power, robustness, and transparency [12]. To solve these problems, we have developed CellSage, a lightweight, powerful CNN architecture specifically tailored to breast cancer classification in histopathological images. The main innovation of CellSage is the combined implementation of three important parts: MSCFE, a multi-scale convolutional feature extractor which includes global tissue context and local cellular morphology; a memory-bound depthwise separable convolutional block which conserves representational power while retaining the burden of computation; and CBAM, which improves map features through attention channel and spatial mechanisms. This attention-enhanced design improves predictive accuracy and improves the model focus on diagnostically relevant regions, interpretability, and clinical trust.

We assess the performance of CellSage on the BreakHis dataset [13], which is one of the most popular datasets for breast cancer histopathology and contains more than 7900 high-resolution images of differing tumor subtypes and magnification levels. After conducting extensive CellSage experiments and comparing it with other advanced architectures, it was observed that CellSage outperformed them in classification accuracy, F1-score, and AUC, alongside requiring many fewer parameters than the other competing models. These results demonstrate the capability of CellSage as a clinically ready, real-time decision support system for digital pathology.

## 2. Related Works

One of the most instructive breakthroughs in computational pathology is the application of deep learning, specifically for the visual recognition tasks performed by the CNNs [14]. For breast cancer diagnosis, CNN-based models have been particularly effective at classifying histopathological images as they automatically construct complex hierarchical features from raw pixels of images [15]. Earlier works utilized off-the-shelf deep CNNs, including VGGNet [16], ResNet [17], and Inception [18], for the binary classification of breast tissue images as either benign or malignant. For instance, ResNet-50 [19] became widely used owing to its residual learning architecture that alleviates the vanishing gradient problem for deep networks [20]. This line of work was expanded further with DenseNet-121, which added dense connectivity between layers to improve feature reuse, thus lessening redundancy in representation learning [21]. Although the described models possess a robust predictive performance, they are resource-intensive and not suitable for clinical use in real time or time-constrained settings due to their hundreds of millions of parameters. As mentioned above, some advanced models use lightweight architectures like MobileNetV2 [22], SqueezeNet [23], and ShuffleNetV2 [24]. These models achieve a smaller model size and lower inference time using depthwise separable convolutions and channel shuffling. This efficiency, unfortunately, results in lower classification accuracy, which is critical in complicated diagnostic processes. Depending on the task, the diagnosis may involve identifying slight shape differences and changes, an intricate problem that is often encountered in cancer cell classification.

Attention mechanisms have been adopted frequently to improve the performance and explainability of CNN-based models [25]. One of the most notable examples is CBAM, which was proposed by Woo et al. [26], capturing attention due to its low weight and efficiency in controlling the focus of the network to salient features. CBAM’s channel and spatial attention are applied sequentially so that the models can focus on diagnostically critical regions while still maintaining low computational cost. It has been applied successfully in several medical image analysis tasks like tumor detection and organ segmentation [27,28]. Though other attention incorporating methods have also been suggested, like the Squeeze-and-Excitation Network (SENet) [29] and self-attention mechanisms based on transformers [30], these tend to add more complexity and strain the system resources.

The most recent additions of the Vision Transformer (ViT) [31] and Swin Transformer [32] have transformed medical image classification. These models enhance context-aware representations and improve global self-attention. In general, the use of these models is only effective with large annotated datasets and powerful computational resources, which, in many circumstances, are lacking within the medical field. Added to this, the heterogeneous nature of histopathological images, including the myriad cell sizes and shapes, as well as differing tissues, makes multi-scale feature extraction a crucial design issue [33]. Feature capture techniques that apply different spatial resolutions include Inception modules and atrous spatial pyramid pooling (ASPP), all of which strive to increase the model spatial feature-finding capabilities [34]. Despite this, the approaches tend to increase model complexity and parameter count, thus creating a balance problem between contextual depth and computational efficiency.

Considering the gaps identified in the current methods regarding the computational burden, the performance of lightweight models, and adaptive attention requirements, the work presented here proposes CellSage. Our model integrates multi-scale convolutional feature extraction, depthwise separable convolutions, and CBAM attention into a single coherent architecture. CellSage is tailored for the classification of histopathological images of breast cancer, providing a noteworthy trade-off between accuracy, interpretability, and ease of deployment. Through this design, we aspire to meet the most pressing demands for high-performance, dependable diagnostic systems in digital pathology.

## 3. Materials and Methods

In this study, we present a novel deep learning model for breast cancer cell classification, specifically designed to address one of the most critical challenges in modern medical diagnostics. Our proposed architecture integrates two key modules: a feature extraction block and a memory-efficient processing block, both of which are followed by CBAM to refine the learned representations and enhance prediction accuracy. The primary objective of this model is to accurately classify cancer cells, a task that remains highly challenging due to the visual similarity between benign and malignant cells. These subtle differences often make diagnosis difficult for the human eye. However, deep learning models have the capability to learn hidden and fine-grained discriminative features, enabling more reliable classification of histopathological images into cancerous and non-cancerous categories.

### 3.1. Convolutional Block Attention Module

The CBAM attention mechanism is a new approach to attention that is lightweight and can enhance CNN structures. It works by augmenting feature representation at the mid-level layer of the CNN architecture using channel and spatial attention. Thus, the network can better emphasize informative features and suppress unhelpful ones. Woo et al. developed CBAM in 2018, and due to its simplicity and efficiency, it has been adopted in multiple vision tasks. The channel attention module (CAM) leverages importance weighting to determine what semantic information needs to be retained. First, spatial information is aggregated using global average pooling and global max pooling, which yield complementary descriptors of the feature map. These descriptors are processed through a shared MLP with subsequent merging through element-wise summation. The resultant output is a set of descriptors that can be used to generate attention using a sigmoid activation function to produce a channel attention map. Applying the antenna allows the original feature map to be recalibrated by applying channel-wise multiplication instead of multiplication. This step improves selectivity by allowing the model precise control over highlighted informative feature channels and even the tunable suppression of less relevant channels. Now, the model addresses the focus question within the spatial domain of the feature map after channel attention has been applied. This is achieved through channel-wise max pooling and average pooling, which reduces the channel information to two spatial descriptors. These descriptors are concatenated along the channel axis and convolved with a 7 × 7 kernel, after which a sigmoid is applied to produce a spatial attention map. This map is then used to perform element-wise multiplication with the input feature map, thereby steering the model to focus on critical regions within the image. Spatial attention in CBAM refers to the process by which the model learns to identify and emphasize the most relevant regions in an image. After the feature maps are refined using channel attention (which focuses on “what” features are important), spatial attention determines “where” in the image those features are most informative. This is achieved by generating an attention map over the spatial dimensions of the image, allowing the model to concentrate on critical areas, such as clusters of abnormal cells or regions with structural distortions.

### 3.2. The Proposed Model

**Figure 1:**
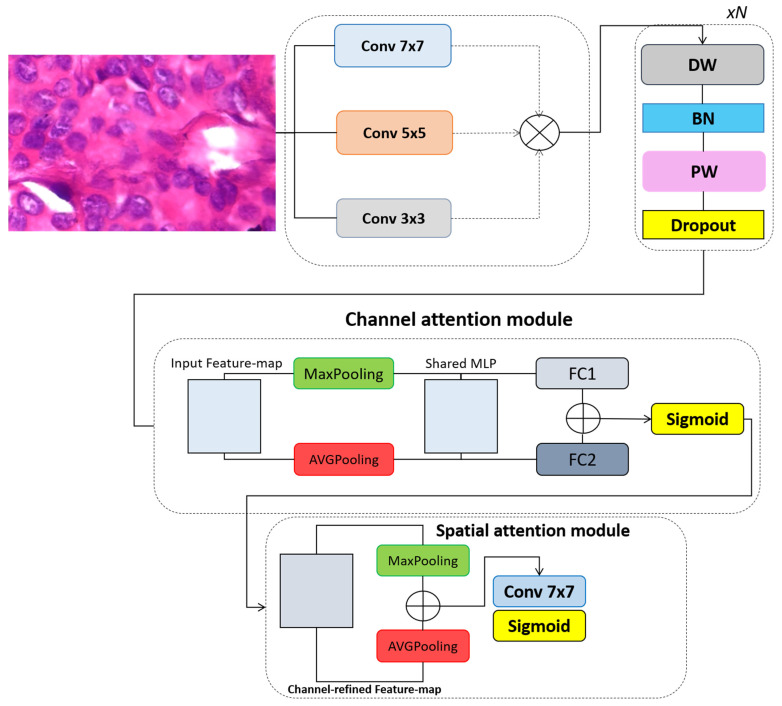
The overall architecture of the proposed CellSage model for breast cancer classification in histopathological images.

In this study, we propose CellSage, a deep learning model developed for the classification of breast cancer in histopathological images (Figure 1). The architecture of CellSage is composed of three sequential components designed to balance diagnostic accuracy with computational efficiency. These include a multi-scale convolutional feature extractor, a depthwise separable convolutional block, and an attention-based refinement module. The input to CellSage is a histological image patch represented as a tensor of size C × H × W, where C denotes the number of color channels (typically 3 for RGB images), and H and W represent the height and width of the image, respectively. The goal is to process this input and output a prediction indicating whether the tissue is benign or malignant. The first component of the model, the multi-scale convolutional feature extractor (MSCFE), is designed to capture both global tissue architecture and local cellular morphology. To achieve this, the input image is passed through three parallel convolutional layers with kernel sizes of 7 × 7, 5 × 5, and 3 × 3. Each of these layers is responsible for learning features at different receptive field sizes, enabling the model to extract a diverse range of structural and textural information. The resulting feature maps from each convolution are concatenated to form a comprehensive multi-scale representation. Following the feature extraction stage, the combined features are processed by a depthwise separable convolutional block, a memory-efficient alternative to standard convolution. This block operates in two steps. First, a depthwise convolution applies a separate filter to each input channel independently, significantly reducing the number of required computations. Next, a pointwise convolution, implemented using 1 × 1 filter, is applied to project the depthwise outputs into a more expressive feature space by combining channel information. To further improve training stability and mitigate overfitting, batch normalization and dropout are applied after the convolutions. This block helps preserve the discriminative power of the extracted features while maintaining a lightweight model profile. To guide the model focus toward the most diagnostically relevant information, we incorporate a Convolutional Block Attention Module (CBAM) as the final refinement stage. CBAM enhances feature representation through two types of attention mechanisms: channel and spatial. The channel attention mechanism determines which feature channels are most important. It achieves this by performing both average pooling and max pooling across the spatial dimensions of each feature map, thereby generating compact descriptors of the image content. These descriptors are passed through a shared multi-layer perceptron (MLP), and the outputs are merged and transformed into a channel attention map using a sigmoid activation function. This map is then multiplied by the input feature map, amplifying the most informative channels. Spatial attention is applied to determine the key regions within the image. This is accomplished by conducting average pooling and max pooling across the channel axis to produce two-dimensional spatial maps. These are concatenated and passed through a convolutional layer with a 7 × 7 kernel to generate a spatial attention map. After applying a sigmoid activation, this map is multiplied by the refined feature map from the channel attention stage, allowing the model to selectively emphasize areas of the image most indicative of malignancy or benignity. The output of this architecture is a probability score indicating the likelihood that the input sample contains malignant features. By combining multi-scale feature extraction, computationally efficient processing, and targeted attention mechanisms, CellSage delivers robust and interpretable predictions while remaining suitable for deployment in real-time diagnostic workflows.

The input image $x^{CxHxW}\in R$ is first processed by the multi-scale convolutional feature extractor (MSCFE), which is designed to capture both broad tissue-level structures and fine-grained cellular features. To achieve this, the module employs parallel convolutional operations with multiple kernel sizes, as described in Equation (1):

(1) $$F_{MSCFE}=Concat(F_{7x7}(x),F_{5x5}(x),F_{3x3}(x))$$

where I denotes the input image patch. The three convolution operations of sizes 7 × 7, 5 × 5, and 3 × 3 are applied in parallel. Each convolution learns different types of spatial features: small kernels focus on local details, while large kernels capture broader structural context. This multi-kernel strategy enables the model to effectively learn from features at varying receptive field sizes, allowing it to extract both coarse contextual patterns and localized textural details essential for accurate cancer classification in histopathological images:

(2) $$F_{DWB}=\;\;Dropout(PW(BatchNorm(DW(F_{MSCFE}))))$$

In Equation (2), we depict the internal structure of the depthwise separable convolution block that comes after MSCFE. Following the inspiration from MobileNet, this block is equipped with depthwise separable convolutions, which, in contrast to standard convolutions, reduce the computational load considerably while maintaining the representation power.

The block contains two main stages: depthwise convolution, performing one filter on every input channel, and channel-wise aggregation via pointwise 1 × 1 convolutions. To improve training stability and convergence, we implement batch normalization after each convolution to normalize feature distributions and incorporate dropout to combat overfitting. This module structure provides a balance between semantic depth and computation efficiency, which is ideal in the analysis of high-resolution medical images:

(3) $$F_{CAM}=\sigma (F_{FC1}(MaxPool(F_{DWB}))+F_{FC2}(AVGPool(F_{DWB})))$$

After DWB, the output feature map is sent to CAM, which is the first part of CBAM. CAM, or the channel attention module, aims to learn “what” the network focuses on and accentuates the response of the most informative channels. CAM uses both global max pooling and global average pooling on the two spatial dimensions of the input feature map to form a feature vector. These two descriptors are passed through a shared MLP model composed of two fully connected layers with one ReLU wholesale layer in between. The outputs from the branches of MLP are then summed in an element-wise fashion to yield a refined channel attention map. This channel attention map is multiplied with the input feature map to adjust the channel-wise responses:

(4) $$F^{\prime }=Multip(F_{CAM}\;,F_{DWB})$$

(5) $$F_{SAM}=\sigma ({Conv}_{7x7}(Concat(MaxPooling(F^{\prime }),AVGPooling(F^{\prime }))))$$

(6) $$F^{\prime \prime }=Multip(F_{SAM}\;,F^{\prime })$$

The second component of the framework is SAM, which focuses on identifying “where” in the spatial domain the model should attend. To construct the spatial attention map, channel-wise max pooling and average pooling are first applied to the feature map refined by CAM. These two spatial descriptors are then concatenated along the channel axis, resulting in a two-channel feature representation. A 7 × 7 convolution is applied to this concatenated map, followed by a sigmoid activation function, producing a spatial attention map that highlights important regions in the feature map. Finally, this attention map is element-wise multiplied with the output from the CAM stage to produce the final refined feature map. The detailed computation steps are formalized in Equations [4,5,6].

## 4. Experiments and Results

In order to evaluate the performance measurement of the implemented CellSage architecture for breast cancer classification using histopathological images, a number of experiments were performed based on the publicly available BreakHis dataset [13]. In this section, we describe the characteristics of the dataset and the corresponding data preprocessing steps, along with the training and testing procedures that were followed for the model evaluation. We performed a comparison of the CellSage model with some other convolutional and transformer models used frequently in the field of medical imaging, subsequently applying the defined metrics. The business metrics considered were classification accuracy, F1-score, and area under the receiver operating characteristic curve (AUC). Additionally, we performed an ablation study to estimate the effect of particular defining structural elements, which include the multi-scale convolutional feature extractor and CBAM. All experiments were conducted to mimic real-life clinical workflows by applying patient-wise data splits to the model training and evaluation, ensuring the assessment truly reflects generalization capabilities. The model performance results discussed in this section demonstrate that CellSage achieves predictive and computational efficiency concurrently, making it suitable for real-time diagnostics.

### 4.1. The Dataset

**Figure 2:**
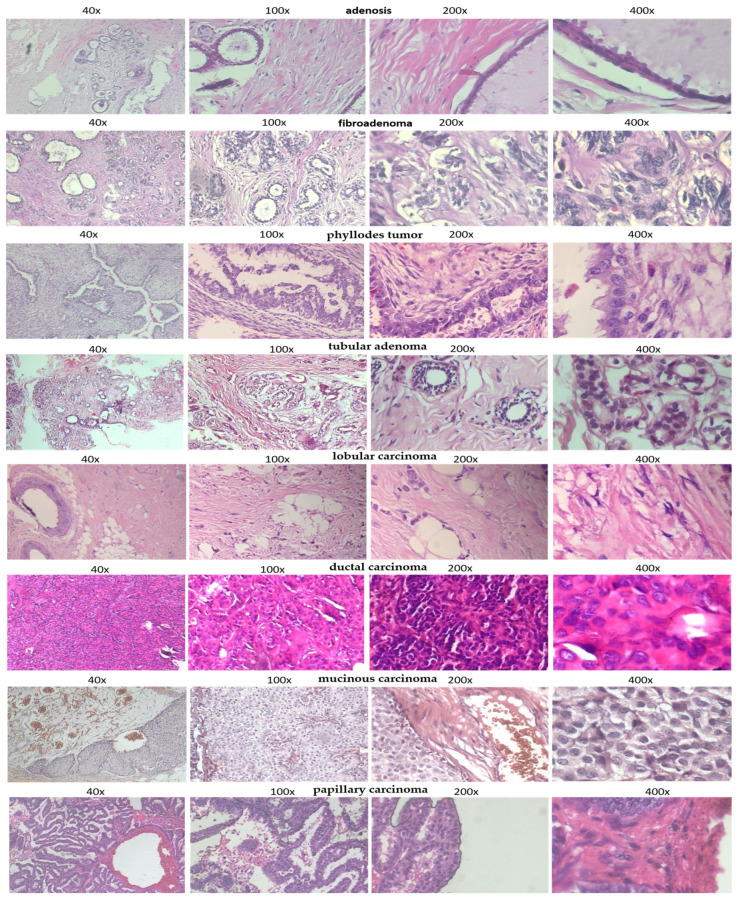
Representative histopathological images from the BreakHis dataset [13] across multiple tumor subtypes and magnification levels (40×, 100×, 200×, and 400×). The top four rows display samples from benign tumor subtypes, including adenosis, fibroadenoma, phyllodes tumor, and tubular adenoma. These tissue structures often exhibit well-differentiated glandular morphology and relatively uniform nuclei. The lower four rows present malignant tumor subtypes, including ductal carcinoma, lobular carcinoma, mucinous carcinoma, and papillary carcinoma. Malignant tissues are characterized by pleomorphic nuclei, disrupted glandular architecture, and increased cellular density. The visual similarity between benign and malignant samples, particularly at higher magnification levels, underscores the diagnostic complexity and motivates the need for robust automated classification methods such as the proposed CellSage.

For assessing the effectiveness of the proposed breast cancer classification model, we focused on utilizing the BreakHis dataset (Breast Cancer Histopathological Image Classification) [13], which is a well-known benchmark in the area of computational pathology. This dataset includes a total of 7909 histopathological images harvested from 82 patients; see Figure 2.

The BreakHis dataset [13] contains a total of 7909 histopathological images collected from 82 patients, spanning multiple tumor subtypes and four magnification levels (40×, 100×, 200×, and 400×). To prevent data leakage and simulate real-world diagnostic conditions, we applied a patient-wise data partitioning strategy, where images from any given patient were included in only one of the three subsets. We used 60% of the patients for training (4744 images), 20% for validation (1580 images), and 20% for testing (1585 images). This ensures a clean evaluation setting where the model must generalize to entirely unseen patient data. In addition, to enhance statistical robustness, we performed 5-fold patient-stratified cross-validation, where the patient-level splits were rotated across folds while maintaining class balance. All reported performance metrics represent the average results across these five folds, with corresponding standard deviations included to reflect variability.

**Figure 3:**
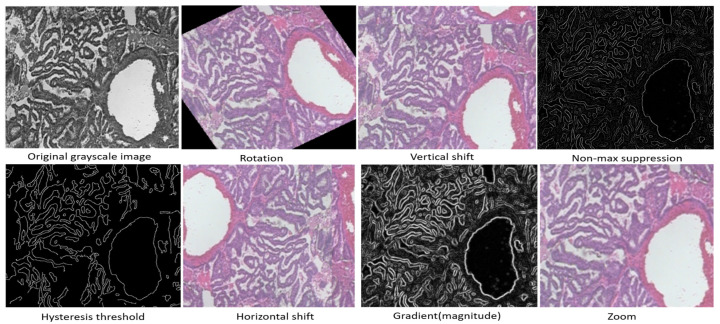
Visualization of data preprocessing techniques applied to histopathological images.

Each tissue specimen was processed with H&E staining and imaged via optical microscopy at four distinct magnification tiers, namely, 40×, 100×, 200×, and 400×, as shown in Figure 1 and Figure 2. The images are stratified into two primary diagnostic categories: tumors that are either benign or malignant. Each of these categories is further subdivided into four specific histological subtypes. The benign class comprises adenosis, fibroadenoma, phyllodes tumor, and tubular adenoma, while the malignant class includes ductal carcinoma, lobular carcinoma, mucinous carcinoma, and papillary carcinoma. Images are in PNG format and have a resolution of around 700 × 460 pixels (Figure 3). One of the most distinguishing features of the BreakHis dataset [13] is high intra-class and inter-class diversity because of differences in tissue architecture, staining methods, and levels of magnification. This heterogeneity greatly complicates automated classification, requiring sophisticated feature-learning methods. In addition, the dataset has some class imbalance, with malignant cases far exceeding benign cases. This discrepancy creates the need for data augmentation and class rebalancing strategies during model training to avoid bias. In this research, we set up the classification task as a binary task: distinguishing histological samples as benign or malignant. To minimize the risk of overfitting and data leakage, a more generalizability-focused approach was employed using patient-level stratified partitioning, where images belonging to a specific patient were allocated solely to the training, validation, or test set. This approach is more suitable for the clinical setting where models are expected to be deployed as they work with data from new patients.

This method is more effective in simulating existing diagnostic frameworks where hidden patient data must be classified correctly. The BreakHis dataset [13] is particularly interesting from a clinical standpoint because it provides a comprehensive and disparate test environment for building reliable cancer detection systems due to its multifaceted tissue presentations as well as extensive control over resolution levels, including minute details. Integrating it into our research gives valid outputs for testing the deep learning model proposed benchmark while allowing reproducible results across different experiments. In Table 1, subtypes are not individually classified in this study; rather, all benign subtypes are grouped under the “benign” label, and all malignant subtypes under “malignant”. However, we include this table to highlight the clinical and morphological complexity inherent in the dataset, which adds depth to the binary classification challenge

**Figure 4:**
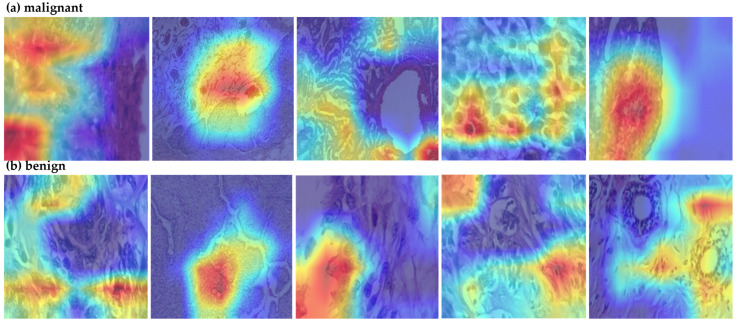
Grad-CAM visualization of malignant tumor; red means high attention and blue means low.

We employed Color Appearance Mapping (CAM) as a stain normalization technique to resolve the inconsistencies in slide preparation, staining, imaging protocols, and image capture technology in relation to color variability, as shown in Figure 4. CAM mitigates the domain shift problem where the color distribution of every input image is mapped to a standard reference stain, thus enhancing visual consistency throughout the dataset. Inaccurate stain normalization compounds the problem of color balancing, which is vital in histopathological assessment. In CAM, diagnostically relevant color details that are subtle but crucial can easily be overshadowed by slight color imbalances that are poorly executed. CAM is performed before dataset alterations are made as an initial preprocessing step. After the stains have been normalized, all pixel values could be rescaled to the [0, 1] interval and standardized using channel-wise image statistics from ImageNet with a mean of [0.485, 0.456, 0.406] and a standard deviation of [0.229, 0.224, 0.225]. Such normalization allows for stable and efficient deep or pre-trained CNN convergence. To increase model robustness concerning both appearance and spatial variability, a more advanced data augmentation procedure was performed during training. Each image underwent a rotation of up to ±30 degrees, horizontal and vertical flips, zoom scaling between 0.8 and 1.2, shifts up to 20 pixels in all directions, and color jittering to simulate stain variability. Collectively, these augmentations model the diversity present in histological images, which mitigates overfitting and encourages the learning of more universal patterns. To eliminate the possibility of data leakage and to maintain a thorough evaluation framework, clear patient-wise partitioning was enforced. All imaging datasets obtained from one patient were kept intact and assigned only to one of the training, validation, or testing subsets. This is more representative of the actual use in the clinical environment, where the model is required to classify data from new, never encountered patients. Due to these preprocessing steps, the final dataset offers greater color consistency and variation in structure, thus enabling the learning of robust and discriminative features critical for dependable cancer cell classification.

All experiments were conducted using PyTorch 2.0 as the deep learning framework, running on an NVIDIA RTX 3090 GPU (24GB VRAM) under CUDA 11.8 (Nvidia Corporation, Santa Clara, CA, USA). The model was trained using the Adam optimizer with an initial learning rate of 1 × 10^−4^, momentum parameters β_1_ = 0.9 and β_2_ = 0.999, and a batch size of 32. We employed a cosine annealing scheduler with warm restarts to dynamically adjust the learning rate and improve convergence. Training was performed for up to 100 epochs, with early stopping enabled (patience = 10) to prevent overfitting. The loss function used was Binary Cross-Entropy with logits (BCEWithLogitsLoss). All weights were initialized using the He normal initialization method. To enhance statistical robustness, we used 5-fold stratified cross-validation at the patient level. Mean performance metrics, along with standard deviation, are reported. Model training, validation, and testing strictly followed non-overlapping patient partitions to simulate real-world deployment scenarios.

### 4.2. Comparative Analysis of Segmentation Models

In Table 2, we have an ablation study examining the impact of several key design elements within the CellSage architecture. The experiments consisted of three model variants: CellSage without CBAM, CellSage without the multi-scale convolutional feature extractor, and the complete CellSage model.

The model without CBAM loses performance drastically, achieving only 92.1% accuracy, an F1 score of 0.90, and an AUC of 0.94. This loss indicates that attention-guided feature refinement is critical for sharpening classification precision and, as such, increases F1 score and AUC. Excluding the multi-scale convolutional block performs worse than model 2 with an accuracy of 91.4%, an F1 score of 0.89, and an AUC of 0.93. These results demonstrate the robust tissue characterization that this model provides and the importance of capturing spatial features at varying resolutions. The full model incorporating both multi-scale feature extraction and CBAM performs best with 94.8% accuracy, an F1 score of 0.93, and an AUC of 0.96. This clearly shows that both modules are essential for optimal performance in classification tasks during image analysis.

In Table 3, the CellSage model has been evaluated against several SOTA deep learning models for medical image classification, specifically for histopathological image classification, and considering various metrics such as classification accuracy, F1-score, AUC, and model parameters (million).

In terms of computational complexity and runtime performance, CellSage demonstrates a highly efficient profile. Despite outperforming larger models such as ResNet-50, DenseNet-121, and ViT in terms of classification accuracy and AUC, CellSage requires only 0.49 GFLOPs per forward pass and maintains an inference time of 8.5 milliseconds per image, which is among the fastest in our benchmark. Additionally, training time per epoch averaged 21 s, making it suitable for fast model development and deployment in real-time systems. These characteristics, coupled with its low parameter count (3.8M), make CellSage an ideal candidate for clinical integration and edge-based histopathological diagnosis.

CellSage performed best with an accuracy of 94.8%, the highest among all models tested. Furthermore, it achieved the best F1 score of 0.93 and AUC of 0.96, showing the best precision–recall trade-off and distinguishing ability between benign and malignant breast cancer tissues. It is also important to note that the parameters count is relatively low compared to CellSage’s competitor, standing at 3.8M, which is low compared to other models and makes CellSage one of the most efficient models in terms of computation (Figure 3). Traditional deep CNNs such as ResNet-50 23.5 M and DenseNet-121 7.9 M show competitive performance with 91.2% and 92.4% accuracy, respectively, but fall short of CellSage both in compactness and predictive strength.

**Figure 5:**
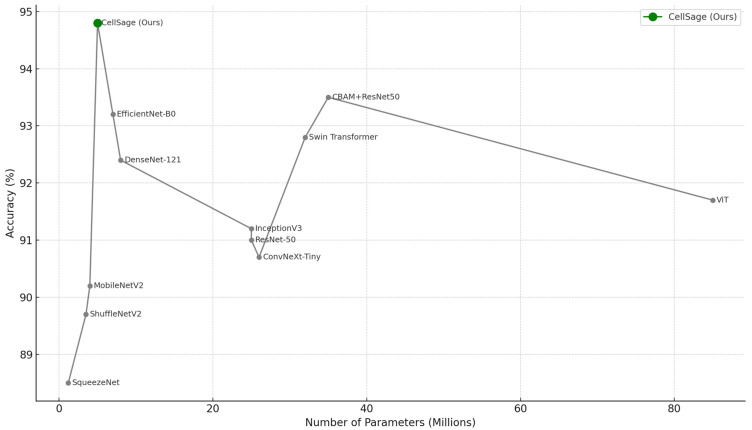
Visualization of the trade-off between model accuracy and parameter count across all compared models. The proposed CellSage model achieves the highest accuracy while maintaining one of the lowest parameters count, demonstrating its suitability for resource-constrained medical applications.

Though lightweight networks MobileNetV2 and ShuffleNetV2 are parameter-efficient with counts of 3.4 M and 2.3 M, and their accuracies of 90.1% and 89.7% are notably low (Figure 5). Transformer-based architectures provide moderate classification results, albeit at a significantly higher cost: ViT 86.0 M and Swin Transformer Tiny 28.0 M. ConvNeXt-Tiny and EfficientNet-B0 claim strong accuracies of 93.7% and 93.3%, respectively, but CellSage outperforms them both in AUC and F1-score, demonstrating the sustained advantage of multi-scale feature extraction with integrated attention.

## 5. Conclusions

In this study, we present CellSage, a lightweight and high-performance deep learning model developed for classifying breast cancer from histopathological images with remarkable accuracy. The proposed architecture incorporates multi-scale convolutional feature extraction, depthwise separable convolutions, and CBAM, which together strike an effective balance between representational power and computational efficiency. Under the substantial challenges posed by varying magnification levels and staining inconsistencies, the model managed to accurately differentiate between benign and malignant subtypes of tissues. CellSage is shown through empirical assessment on the BreakHis dataset [13] to surpass numerous recently published CNN and transformer-based models on almost every metric, such as overall accuracy, F1-score, and AUC, and most importantly, with a far lower parameter count. This further emphasizes the strength achieved by applying a design philosophy that favors lightweight models coupled with adaptive attention mechanisms in the context of enhancing predictive accuracy in medical imaging while improving interpretability. In addition, the implementation of exhaustive data augmentation techniques along with stain normalization increases the model robustness while enhancing its generalizability, thus making it ready for actual clinical use. The rigorous patient-level data partitioning methodology used in our evaluation scheme guarantees that the stated performance measures capture the true ability of the model to generalize to previously unseen data from new patients. Future investigations could expand CellSage to multi-class classification problems, incorporate histopathological grading, or assess domain adaptation across heterogeneous datasets with differing staining protocols. Furthermore, integrating CellSage with tools of explainable AI could dramatically improve trust and adoption in clinical settings. In any case, CellSage is a major step toward scalable and interpretable cancer diagnostic tools that require minimal resources in digital pathology. Despite its strong performance, the proposed CellSage model has limitations. The model is currently applied at the patch level, rather than on full whole-slide images (WSIs), which restricts its immediate applicability to large-scale diagnostic pipelines.

## Figures and Tables

**Table 1:** Overview of the histopathological subtypes present in the BreakHis dataset [13]. While the proposed model performs binary classification (benign vs. malignant), this table illustrates the underlying subtype diversity within each group, which contributes to the visual and structural variability the model must learn to generalize across.

**Benign:**	
adenosis	Overgrowth of glands in the breast lobules
fibroadenoma	Common benign breast tumor made of glandular and fibrous tissues
phyllodes tumor	Rare fibroepithelial tumor, usually benign
tubular adenoma	Rare, benign glandular tumor
**Malignant:**	
ductal carcinoma	Most common invasive breast cancer, begins in ducts
lobular carcinoma	Starts in the lobules (milk-producing glands)
mucinous carcinoma	Tumor made mostly of mucus-producing cancer cells
papillary carcinoma	Rare subtype, finger-like projections under microscope

**Table 2:** The partial combination of the blocks and full model.

Model Variant	Accuracy (%)	F1 Score	AUC
CellSage w/o CBAM	92.1	0.90	0.94
CellSage w/o Multi-Scale Conv	91.4	0.89	0.93
CellSage Full (Ours)	94.8	0.93	0.96

**Table 3:** The comparison results of the SOTA models.

Model	Accuracy (%)	F1 Score	AUC	Params (M)	FLOPs (G)	Inference Time (ms)	Training Time/Epoch (s)
ResNet-50	91.2	0.89	0.93	23.5	4.1	15.8	36
CBAM + ResNet-50	93.7	0.92	0.95	24.1	4.3	18.2	39
DenseNet-121	92.4	0.91	0.94	7.9	2.9	13.1	33
MobileNetV2	90.1	0.88	0.91	3.4	0.31	9.3	17
EfficientNet-B0	93.3	0.92	0.95	5.3	0.39	10.2	22
ViT	91.8	0.89	0.92	86.0	16.8	39.5	65
InceptionV3	91.5	0.90	0.94	23.9	5.7	16.4	38
ShuffleNetV2	89.7	0.87	0.90	2.3	0.28	8.8	16
SqueezeNet	88.5	0.85	0.89	1.2	0.26	8.1	15
ConvNeXt-Tiny	90.7	0.89	0.91	24.6	4.8	20.1	40
Swin Transformer	92.9	0.91	0.94	28.0	6.2	22.7	45
CellSage (Ours)	94.8	0.93	0.96	3.8	0.49	8.5	21

## Data Availability

All used datasets are available online through open access.
